# Long-term glycemic variability predicts compromised development of heart failure with improved ejection fraction: a cohort study

**DOI:** 10.3389/fendo.2023.1211954

**Published:** 2023-09-20

**Authors:** Chen Die Yang, Jia Wei Chen, Jin Wei Quan, Xin Yi Shu, Shuo Feng, Muladili Aihemaiti, Feng Hua Ding, Wei Feng Shen, Lin Lu, Rui Yan Zhang, Xiao Qun Wang

**Affiliations:** ^1^Department of Cardiovascular Medicine, Ruijin Hospital, Shanghai Jiao-Tong University School of Medicine, Shanghai, China; ^2^Institute of Cardiovascular Disease, Shanghai Jiao-Tong University School of Medicine, Shanghai, China

**Keywords:** glycemic variability, heart failure with improved ejection fraction, heart failure with reduced ejection fraction, myocardial recovery, fasting plasma glucose

## Abstract

**Background:**

A substantial portion of heart failure (HF) patients adherent to guideline-directed medical therapies have experienced improved ejection fraction (EF), termed HFimpEF. Glycemic variability (GV) has emerged as a critical cardiometabolic factor. However, the relation between long-term GV and the incidence of HFimpEF is still unclear.

**Methods:**

A total of 591 hospitalized HF patients with reduced EF (HFrEF, EF≤ 40%) admitted from January 2013 to December 2020 were consecutively enrolled. Repeat echocardiograms were performed at baseline and after around 12 months. The incidence of HFimpEF, defined as (1) an absolute EF improvement ≥10% and (2) a second EF > 40% and its association with long-term fasting plasma glucose (FPG) variability were analyzed.

**Results:**

During a mean follow-up of 12.2 ± 0.6 months, 218 (42.0%) patients developed HFimpEF. Multivariate analysis showed FPG variability was independently associated with the incidence of HFimpEF after adjustment for baseline HbA1c, mean FPG during follow-up and other traditional risk factors (odds ratio [OR] for highest vs. lowest quartile of CV of FPG: 0.487 [95% CI 0.257~0.910]). Evaluation of GV by alternative measures yielded similar results. Subgroup analysis revealed that long-term GV was associated with HFimpEF irrespective of glycemic levels and diabetic conditions.

**Conclusions:**

This study reveals that greater FPG variability is associated with compromised development of HFimpEF. A more stable control of glycemic levels might provide favorable effects on myocardial functional recovery in HF patients even without diabetes.

## Introduction

Heart failure (HF) is a prevalent clinical syndrome with high mortality and morbidity. With the development of guideline-directed medical treatment and device therapy, a substantial proportion of HF patients with reduced ejection fraction (EF, HFrEF) have experienced improved left ventricular (LV) EF, thereafter termed HF with recovered or improved EF (HFimpEF) (1–5). Compared with other types of HF, HFimpEF possesses distinct pathophysiological characteristics, clinical manifestations, and better prognosis (4–7). In the 2022 AHA/ACC/HFSA Guideline for the Management of HF (8), HFimpEF was thus proposed as a new classification of HF. The process of myocardial functional improvement is coordinately driven by adaptive molecular change, metabolic profile alteration, improved cardiomyocyte contractility and LV geometric restoration (7, 9, 10). However, the predisposing factors for HFimpEF are still under investigation.

Glycemic variability (GV) refers to fluctuations in glucose levels within-days or over months to years. GV has been recognized as a critical risk factor for diabetic macrovascular and microvascular complications (11–14), and adverse cardiovascular events even in patients without diabetes (15–21). In the setting of acute HF, elevated in-hospital GV confers higher risk of both short-term and long-term mortality in addition to classic glucose metrics (22, 23). The adverse impact of long-term glucose fluctuations on clinical outcomes has also been confirmed in chronic HF patients (24, 25). Nevertheless, the impact of GV on myocardial recovery in failing hearts is still unclear. In the present study, we analyzed the relationship between long-term GV and the incidence of HFimpEF.

## Methods

### Study population

**Figure 1:**
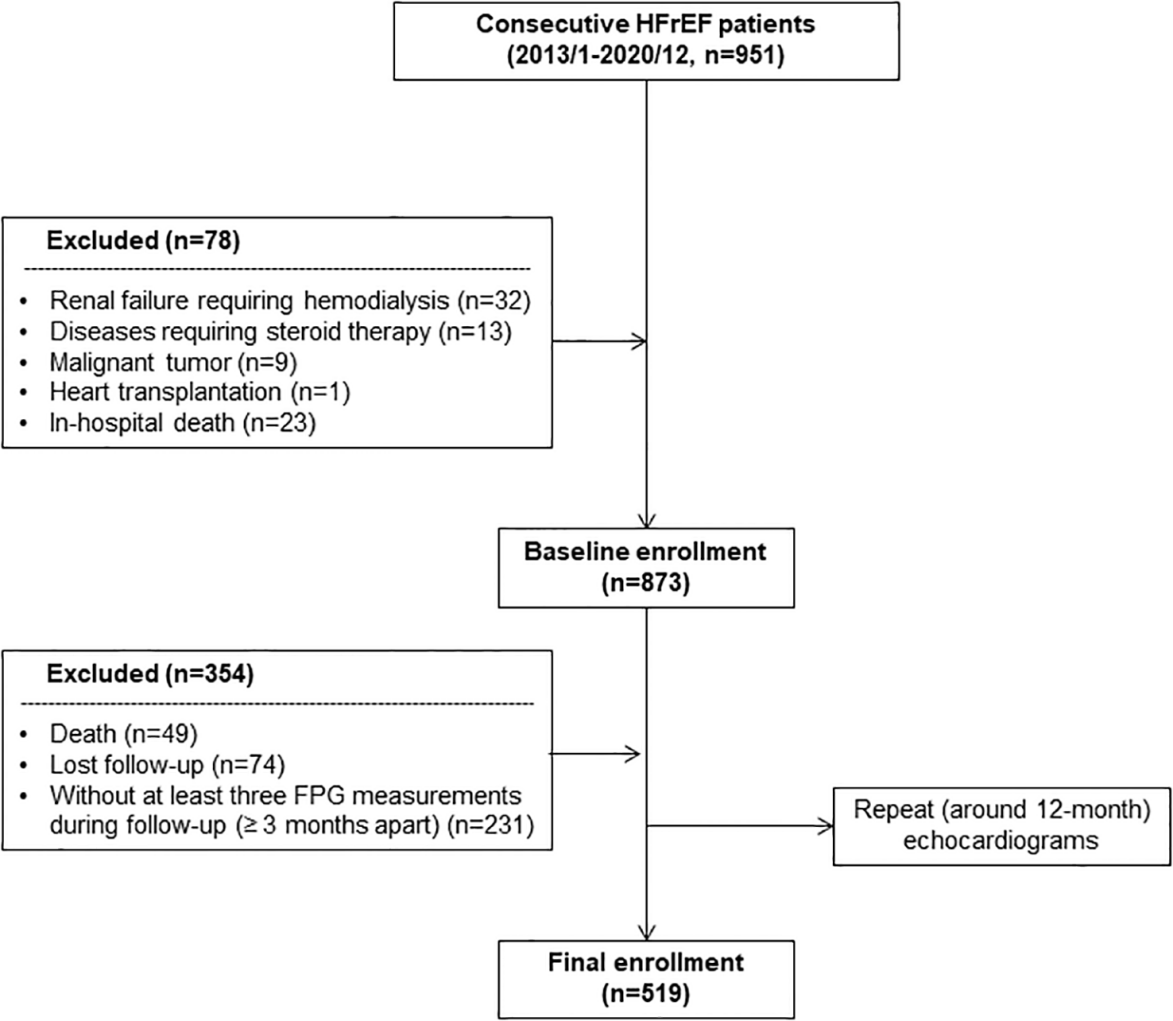
Flowchart of patient enrollment. FPG, fasting plasma glucose; HFrEF, heart failure with reduced ejection fraction.

We consecutively enrolled 951 patients diagnosed with HFrEF (EF ≤ 40%) on hospitalization between January 2013 and December 2020 in Shanghai Ruijin Hospital. A total of 78 patients comorbid with renal failure requiring hemodialysis (n=32), diseases requiring steroid therapy (n=13), malignant tumor (n=9), heart transplantation (n=1) and in-hospital death (n=23) were excluded. The enrolled patients were routinely followed up and underwent repeat echocardiograms at around 12-month ( ± 1 month). During follow-up, there were 49 patients who died for any reason within 13 months from the index admission date and thus were excluded. Another 74 subjects were also excluded due to loss to echocardiogram follow-up for any other reason. Given that the development of HFimpEF was the primary endpoint, patients who received the follow-up echocardiogram at around 12-month but died thereafter were not excluded from the analysis. For calculation of long-term GV, subjects (n=231) without at least three fasting plasma glucose (FPG) measurements with ≥3 months apart were further excluded (**Figure 1**).

The primary outcome was the development of HFimpEF, which was diagnosed based on follow-up echocardiogram according to the universal HFimpEF definition (26): (1) an absolute EF improvement ≥10% and (2) a second EF > 40%.

This study complies with the Declaration of Helsinki. The study protocol was approved by Shanghai Ruijin Hospital ethics committee, and written informed consent was obtained from all participants.

### Clinical and biochemical assessments

Detailed information of medical history and lifestyle was obtained using a standard questionnaire by trained physicians on admission. Body mass index (BMI) was calculated as weight/height^2^ (kilograms per square meter). Body surface area (BSA) was calculated by Stevenson’s formula: 0.0061 × height + 0.0128 × weight - 0.1529 (27). Hypertension was diagnosed according to the seventh report of the Joint National Committee on prevention, detection, evaluation, and treatment of high blood pressure (BP; JNC 7) (28). The diagnosis of diabetes was made according to the criteria of American Diabetes Association (29). Ischemic etiology was diagnosed based on medical history survey, examination by coronary computed tomography angiography (CTA) or coronary angiogram.

All the blood samples were drawn after overnight fasting. Plasma glucose, insulin, liver and renal function, total cholesterol, low-density lipoprotein (LDL) cholesterol, high-density lipoprotein (HDL) cholesterol, triglycerides, and N-terminal pro-B-type natriuretic peptide (NT-proBNP) were assessed (HITACHI 912 Analyzer, Roche Diagnostics, Germany). The estimated glomerular filtration rate (eGFR) was computed using the Chronic Kidney Disease Epidemiology Collaboration equation (30). Blood HbA1c was measured using ion-exchange high performance liquid chromatography with Bio-rad Variant Hemoglobin Testing System (Bio-Rad Laboratories, USA).

### Echocardiographic examination

Comprehensive transthoracic echocardiography was performed using a commercially available system (Vivid-I, GE Healthcare, Milwaukee, WI). The sonographers were blinded to this study. Two-dimensional echocardiography and Doppler flow imaging were recorded from standard parasternal and apical transducer positions.

EF was calculated using the modified Simpson’s biplane technique. The LV length was measured in an apical 4-chamber view. To facilitate application of clinical normality cut points, LV end-diastolic volume (EDV) and end-systolic volume (ESV) were indexed by BSA calculated at the study time point. LV mass was estimated from M-mode measurements by the formula: LV mass = $0.8\times 1.04\times \left[{\left(EDD+IVST+PWT\right)}^{3}-EDD^{3}\right]+0.6$ , and was indexed by BSA, where EDD is LV end-diastolic diameter, IVST is interventricular septal thickness, PWT is LV posterior wall thickness.

### GV measurement

Long-term GV was measured during follow-up period for ≥ 3 times with at least 3-month intervals. The mean and variability of FPG were calculated. FPG variability was primarily defined as intraindividual coefficient of variation (CV) of FPG across visits, which was calculated as the standard deviation (SD) divided by the mean value. The alternative variability of FPG includes: 1) average successive variability (ASV), which was defined as the average absolute difference between successive values and 2) the variability independent of the mean (VIM), which was calculated by the equation as previously reported (19): VIM=100×SD/mean^β^, where β is the regression coefficient based on natural logarithm of SD on natural logarithm of mean of the study population. FPG variability was calculated both as continuous and categorical variables grouped by quartiles of CV, ASV or VIM.

### Nested case-control study

A case-control study was nested into the HF cohort to examine the association between GV and the development of HFimpEF. Each case (HFimpEF) was matched by 1 control (persistent HFrEF) randomly sampled from the cohort members based on sex, age ( ± 2 years) and duration of echocardiogram follow-up. Meanwhile, GV was treated as a dichotomized variable by fusing the original quartile 1~2 as stable glycemic control and quartile 3~4 as unstable glycemic control of all the 3 GV measures (CV, ASV, VIM). A total of 200 case-control pairs were matched for the final analysis.

### Statistical analysis

Continuous variables were presented as median (interquartile range [IQR]) or mean ± SD, and categorical data were summarized as frequencies (percentages). Normal distribution of continuous variables was evaluated by Shapiro-Wilk test. For normally distributed variables, differences in quartiles of FPG variability and subgroup analysis were performed by one-way analysis of variance (ANOVA) followed by *post hoc* Bonferroni correction. For non-normally distributed continuous variables, differences were analyzed by Mann-Whitney U test or Kruskal-Wallis test. Differences in categorical variables were analyzed by χ^2^ test. Univariate logistic regression analysis was performed to identify predictors for HFimpEF. Afterwards, multivariate regression models were constructed to interrogate the association between FPG variability and HFimpEF. In model 1, age and sex were adjusted. In model 2, additional adjustment was performed with HF etiology, BP, BMI, and history of diabetes. In model 3, we further adjusted HbA1c, renal function, mean FPG levels during follow-up and baseline EDV index. In model 4, cardiac resynchronizing therapy (CRT) and medical therapies including beta-blockers, angiotensin converting enzyme inhibitors (ACEI), angiotensin receptor blockers (ARB), angiotensin receptor-neprilysin inhibitors (ARNI), spironolactones as well as sodium-glucose cotransporter 2 (SGLT2) inhibitors were additionally adjusted. FPG variability was analyzed both as continuous and categorical variables in univariate and multivariate regression models. The association between GV and HFimpEF in the nested case-control study was analyzed by conditional logistic regression.

All statistical analyses were performed using the R statistical package v.4.0.3 (R Project for Statistical Computing, Vienna, Austria). A 2-tailed *P*<0.05 was considered statistically significant.

## Results

### Baseline characteristics of the study population

A total of 519 HFrEF patients were finally enrolled in this study. The mean age was 61.3 ± 12.7 years with 80.3% male patients. Among these subjects, 30.1% were with diabetes (n=156). There were 53.2% of HFrEF patients with an ischemic etiology, and 83.3% of them were diagnosed based on coronary CTA or angiogram during the index admission. The mean number of intrapersonal FPG tests was 5.34 ± 2.47 times. The mean FPG level during follow-up was 6.61 ± 1.99 mmol/L, and CV, ASV, VIM of FPG during follow-up were 0.162 [IQR 0.093~0.268], 1.190 [IQR 0.568~2.235] and 0.641 [IQR 0.408~0.968], respectively. Correlation analyses showed that GV indices such as CV and ASV of FPG were positively correlated to mean FPG levels (CV: Pearson’s r = 0.56, *P*<0.001; ASV: Pearson’s r= 0.73, *P*<0.001), whereas no correlation was found between VIM of FPG and mean FPG levels (Pearson’s r= -0.04, *P*=0.348).

After dividing these patients into 4 groups based on quartiles of CV of FPG, we found subjects with higher GV tended to be elder, more frequently with diabetes and ischemic HF etiology, and with higher levels of baseline HbA1c, FPG as well as NT-proBNP. Subjects in the highest quartile were more frequently on anti-platelet therapy. There was no significant difference in sex, BMI, smoking habits, history of hypertension, atrial fibrillation, New York Heart Association (NYHA) grades, lipid profiles, renal function, CRT implantation and other therapies between the 4 quartiles **(** **Table 1****)**.

**Table 1:** Baseline demographic and clinical characteristics.

Quartiles of CV of FPG	Q1<0.093	Q2 0.094~0.162	Q3 0.163~0.268	Q4 ≥0.269	*P*-value
n	130	130	129	130	
Demographic characteristics and clinical assessments					
Male sex	112 (86.2)	98 (75.4)	104 (80.6)	103 (79.2)	0.178
Age, years	59.75 ± 13.31	60.28 ± 13.05	61.22 ± 12.00	64.06 ± 12.15	0.030
Diabetes	20 (15.4)	19 (14.6)	45 (34.9)	72 (55.4)	<0.001
Hypertension	65 (50.0)	62 (47.7)	71 (55.0)	77 (59.2)	0.243
Atrial fibrillation	16 (12.3)	20 (15.4)	14 (10.9)	10 (7.7)	0.273
Dyslipidemia	80 (61.5)	70 (53.8)	78 (60.5)	85 (65.4)	0.290
Smoking habits	60 (46.2)	50 (38.5)	45 (34.9)	54 (41.5)	0.297
BMI, kg/m^2^	24.77 ± 3.43	24.79 ± 3.87	24.90 ± 3.74	24.42 ± 3.54	0.754
Systolic BP, mmHg	120.56 ± 20.44	127.67 ± 24.79	125.16 ± 19.97	123.83 ± 21.21	0.074
Diastolic BP, mmHg	71.81 ± 13.34	77.97 ± 18.40	76.16 ± 14.19	75.68 ± 15.08	0.015
Ischemic etiology	60 (46.2)	58 (44.6)	73 (56.6)	85 (65.4)	0.002
NYHA grades (II/III/IV)	27 (20.8)/ 80 (61.5)/ 23 (17.7)	26 (20.0)/ 88 (67.7)/ 16 (12.3)	21 (16.3)/ 85 (65.9)/ 23 (17.8)	27 (20.8)/ 85 (65.4)/ 18 (13.8)	0.775
Laboratory measurements					
HbA1c, %	6.02 ± 0.80	6.13 ± 0.84	6.48 ± 1.15	.24 ± 1.55	<0.001
FPG, mmol/L	5.31 (4.87~5.92)	5.26 (4.77~6.06)	5.78 (4.90~7.31)	6.59 (5.14~9.61)	<0.001
Triglyceride, mmol/L	1.35 (0.99~1.90)	1.30 (0.94~1.63)	1.27 (0.95~1.66)	1.27 (0.94~1.69)	0.446
Total cholesterol, mmol/L	4.14 ± 1.08	4.00 ± 1.04	4.02 ± 1.22	3.98 ± 1.11	0.644
HDL cholesterol, mmol/L	1.08 ± 0.26	1.07 ± 0.28	1.02 ± 0.27	1.01 ± 0.28	0.052
LDL cholesterol, mmol/L	2.51 ± 0.86	2.46 ± 0.88	2.44 ± 0.97	2.42 ± 0.93	0.879
eGFR, mL/min/1.732m^2^	90.75 ± 16.97	87.96 ± 16.99	87.75 ± 24.01	84.81 ± 22.32	0.136
NT-proBNP, pg/mL	1674.0 (714.3~3237.0)	2166.0 (588.9~4190.5)	2866.0 (1360.0~5075.0)	3598.5 (1695.5~7174.8)	<0.001
CRT implantation					
CRT	16 (12.3)	22 (16.9)	13 (10.1)	12 (9.2)	0.227
Medication					
Aspirin	70 (53.8)	67 (51.5)	73 (56.6)	71 (54.6)	0.877
P_2_Y_12_ inhibitors	48 (36.9)	53 (40.8)	64 (49.6)	77 (59.2)	0.001
Beta-blockers	119 (91.5)	111 (85.4)	117 (90.7)	106 (81.5)	0.051
ACEI/ARB	61 (46.9)	71 (54.6)	61 (47.3)	71 (54.6)	0.402
ARNI	45 (34.6)	36 (27.7)	36 (27.9)	25 (19.2)	0.051
SGLT2 inhibitors	7 (5.4)	9 (6.9)	10 (7.8)	6 (4.6)	0.713
Spironolactones	104 (80.0)	94 (72.3)	86 (66.7)	86 (66.2)	0.048
Diuretics	82 (63.1)	82 (63.1)	86 (66.7)	83 (63.8)	0.921
Statins	64 (49.2)	69 (53.1)	72 (55.8)	76 (58.5)	0.485

ACEI, angiotensin-converting enzyme inhibitors; ARB, angiotensin receptor blockers; ARNI, angiotensin-receptor neprilysin inhibitors; BMI, body mass index; BP, blood pressure; CRT, cardiac resynchronizing therapy; CV, coefficient of variation; eGFR, estimated glomerular filtration rate; FPG, fasting plasma glucose; HDL, high-density lipoprotein; LDL, low-density lipoprotein; NT-proBNP, N-terminal pro-B-type natriuretic peptide; NYHA, New York Heart Association; SGLT2, sodium-glucose cotransporter 2.

In addition, documented hypoglycemic event defined as FPG< 2.8 mmol/L during follow-up was compared. In our study, 3.3% of the subjects suffered hypoglycemic episodes, which was more frequent in higher quartiles of CV of FPG (0 vs. 0 vs. 2.3% vs. 10.8%, *P*<0.003).

### Changes in LV geometry and function

LV geometric and functional parameters at baseline and around 12-month follow-up were compared in subjects with different quartiles of CV of long-term FPG **(** **Table 2****)**. At baseline, there was no significant difference in LV function and volumes. During the follow-up, EF was improved from 32.38% ± 5.28% to 42.12% ± 10.17% (*P*<0.001) and LV volumes were restored in the overall population. However, the trend towards EF improvement was markedly impaired with increasing FPG variability (*P*=0.003). LV reverse remodeling was also attenuated in patients with high FPG variability (ΔEDV index: *P*=0.004; ΔESV index: *P*<0.001).

**Table 2:** Left ventricular geometric and functional changes during follow-up.

Quartiles of CV of FPG		Q1<0.093	Q2 0.094~0.162	Q3 0.163~0.268	Q4 ≥0.269	*P*-value
EDV index, mL/m^2^	B	125.93 ± 31.87	123.05 ± 29.95	120.49 ± 31.84	116.79 ± 33.44	0.140
	F	107.60 ± 30.91	104.09 ± 29.03	109.33 ± 35.64	108.14 ± 33.07	0.608
	Δ	-18.33 ± 25.44	-18.96 ± 25.29	-11.16 ± 23.22	-8.65 ± 32.39	0.004
ESV index, mL/m^2^	B	83.50 ± 24.59	81.67 ± 24.37	78.26 ± 26.16	76.12 ± 30.84	0.125
	F	60.56 ± 26.86	58.70 ± 25.22	64.31 ± 31.43	64.44 ± 28.76	0.285
	Δ	-22.94 ± 23.50	-22.97 ± 23.37	-13.95 ± 22.36	-11.68 ± 31.12	<0.001
EDD, mm	B	65.61 ± 7.62	64.92 ± 6.99	64.02 ± 8.11	62.65 ± 7.44	0.011
	F	61.04 ± 8.16	60.18 ± 7.30	61.49 ± 8.96	60.33 ± 8.03	0.529
	Δ	-4.57 ± 6.17	-4.75 ± 6.20	-2.53 ± 5.66	-2.32 ± 7.36	0.001
ESD, mm	B	54.45 ± 7.57	53.95 ± 7.00	52.65 ± 8.54	51.51 ± 7.84	0.010
	F	47.14 ± 9.26	46.33 ± 8.41	48.30 ± 10.14	47.61 ± 9.12	0.376
	Δ	-7.32 ± 7.68	-7.62 ± 7.77	-4.35 ± 7.71	-3.90 ± 9.29	<0.001
IVST, mm	B	9.28 ± 1.62	9.32 ± 1.46	9.27 ± 1.48	9.35 ± 1.78	0.971
	F	9.58 ± 1.57	9.62 ± 1.48	9.48 ± 1.42	9.70 ± 1.82	0.730
	Δ	0.31 ± 1.29	0.30 ± 1.21	0.21 ± 1.36	0.35 ± 1.54	0.871
PWT, mm	B	9.00 ± 1.36	9.05 ± 1.47	8.99 ± 1.22	9.02 ± 1.43	0.985
	F	9.07 ± 1.19	9.29 ± 1.34	9.00 ± 1.27	9.05 ± 1.27	0.260
	Δ	0.07 ± 1.32	0.24 ± 1.29	0.01 ± 1.20	0.04 ± 1.37	0.482
LV mass index, g/m^2^	B	146.24 ± 38.55	143.71 ± 33.51	140.19 ± 34.72	138.69 ± 37.64	0.349
	F	131.37 ± 31.32	130.59 ± 29.89	132.64 ± 37.01	132.72 ± 35.28	0.950
	Δ	-14.86 ± 31.17	-13.12 ± 29.65	-7.55 ± 29.34	-5.96 ± 27.14	0.050
EF, %	B	32.26 ± 4.94	32.23 ± 5.01	32.20 ± 5.48	32.83 ± 5.70	0.739
	F	43.48 ± 9.73	43.76 ± 10.03	40.74 ± 10.10	40.47 ± 10.49	0.009
	Δ	11.22 ± 9.36	11.53 ± 10.21	8.53 ± 9.54	7.64 ± 11.47	0.003

B, baseline; F, follow-up; Δ, changes in corresponding parameters; CV, coefficient of variation; EDD, end-diastolic diameter; EDV, end-diastolic volume; EF, ejection fraction; ESD, end-systolic diameter; ESV, end-systolic volume; FPG, fasting plasma glucose; IVST, interventricular septal thickness; LV, left ventricle; PWT, posterior wall thickness.

### Association between FPG variability and HFimpEF

After 12.2 ± 0.6 months, 218 (42.0%) patients developed HFimpEF and another 301 (58.0%) patients remained HFrEF.

Univariate regression analysis **(** **Supplementary Table 1****)** revealed that predictors for HFimpEF were younger age, non-diabetes, non-ischemic etiology, higher BP, lower HbA1c levels, lower EDV index and use of SGLT2 inhibitors. The 3 measures of FPG variability (CV, ASV and VIM) were all inversely associated with HFimpEF either when treated as continuous or categorical variables.

Multivariate regression analysis **(** **Table 3****)** showed that different measures of FPG variability were persistently associated with the development of HFimpEF after adjustment for age and sex (Model 1), clinical characteristics (Model 2), renal function, baseline HbA1c, mean FPG control levels, LV volumes (Model 3) and treatment regimens (Model 4). In the full adjustment model (Model 4), patients with highest quartile of CV of FPG corresponded to a 51.3% (OR: 0.487 [95% CI 0.257~0.910]) decreased likelihood of HFimpEF as compared to the lowest quartile. Similar findings were also observed when these measures of FPG variability were treated as continuous variables **(** **Supplementary Table 2****)**.

**Table 3:** Multivariate regression analysis for development of HFimpEF.

	Model 1		Model 2		Model 3		Model 4	
	OR (95% CI)	*P*	OR (95% CI)	*P*	OR (95% CI)	*P*	OR (95% CI)	*P*
CV of FPG		0.007^*^		0.014^*^		0.007^*^		0.011^*^
Q1 (<0.093)	Reference	–	Reference	–	Reference	–	Reference	–
Q2 (0.093~0.162)	1.114 (0.678~1.831)	0.670	0.994 (0.598~1.651)	0.981	0.960 (0.571~1.610)	0.876	0.931 (0.550~1.573)	0.788
Q3 (0.163~0.268)	0.656 (0.395~1.082)	0.100	0.628 (0.373~1.052)	0.078	0.578 (0.337~0.985)	0.045	0.589 (0.341~1.011)	0.056
Q4 (≥0.269)	0.565 (0.337~0.940)	0.029	0.558 (0.321~0.962)	0.037	0.478 (0.256~0.883)	0.019	0.487 (0.257~0.910)	0.025
ASV of FPG		0.020^*^		0.044^*^		0.017^*^		0.015^*^
Q1 (<0.568)	Reference	–	Reference	–	Reference	–	Reference	–
Q2 (0.568~1.190)	0.715 (0.434~1.174)	0.185	0.627 (0.375~1.042)	0.073	0.614 (0.363~1.033)	0.067	0.618 (0.362~1.048)	0.075
Q3 (1.191~2.240)	0.618 (0.372~1.022)	0.062	0.604 (0.356~1.021)	0.061	0.565 (0.324~0.980)	0.043	0.541 (0.308~0.946)	0.032
Q4 (≥2.241)	0.557 (0.334~0.921)	0.023	0.561 (0.321~0.976)	0.041	0.446 (0.224~0.875)	0.020	0.442 (0.219~0.881)	0.021
VIM of FPG		0.011^*^		0.015^*^		0.013^*^		0.018^*^
Q1 (<0.408)	Reference	–	Reference	–	Reference	–	Reference	–
Q2 (0.408~0.641)	0.673 (0.408~1.107)	0.120	0.635 (0.381~1.054)	0.080	0.622 (0.369~1.043)	0.073	0.633 (0.373~1.069)	0.088
Q3 (0.642~0.968)	0.679 (0.412~1.115)	0.127	0.667 (0.401~1.107)	0.119	0.637 (0.378~1.067)	0.088	0.660 (0.388~1.116)	0.122
Q4 (≥0.969)	0.497 (0.297~0.825)	0.007	0.500 (0.296~0.837)	0.009	0.487 (0.283~0.829)	0.009	0.496 (0.287~0.851)	0.011

Model 1, adjustment for age and sex.

Model 2, additional adjustment for HF etiology, systolic and diastolic blood pressure, body mass index and history of diabetes.

Model 3, additional adjustment for HbA1c, renal function, mean fasting glucose levels during follow-up, and baseline left ventricular end-diastolic volume index.

Model 4, additional adjustment for CRT, use of beta-blockers, angiotensin converting enzyme inhibitors, angiotensin receptor blockers, angiotensin receptor-neprilysin inhibitors, spironolactones, sodium-glucose cotransporter 2 inhibitors.

ASV, average successive variability; CI, confidence interval; CRT, cardiac resynchronizing therapy; CV, coefficient of variation; FPG, fasting plasma glucose; HF, heart failure; HFimpEF, heart failure with improved ejection fraction; OR, odds ratio; VIM, variability independent of the mean.

^*^P for trend.

**Figure 2:**
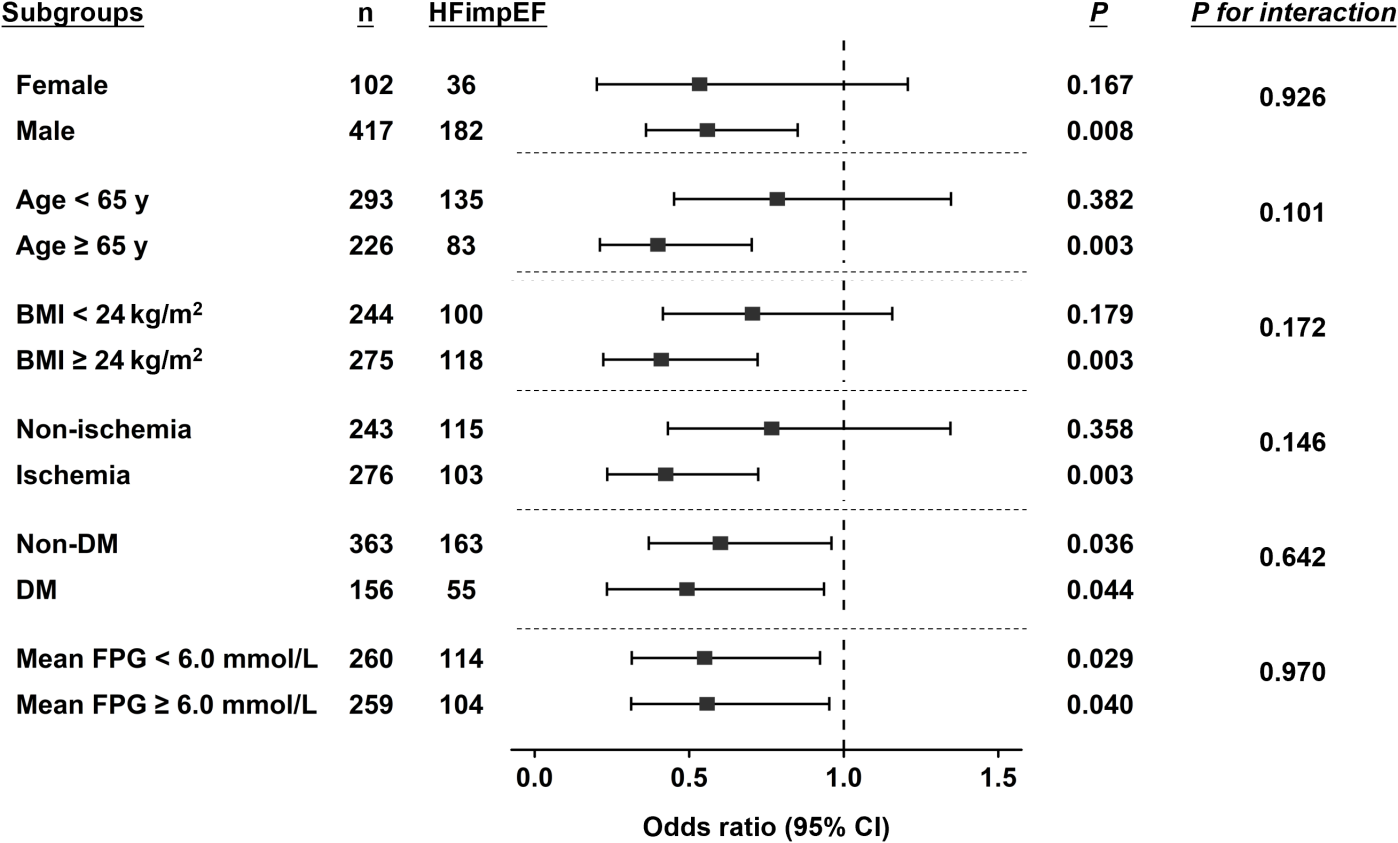
Subgroup analysis by forest plot. Forest plot shows the association between VIM of long-term FPG and incidence of HFimpEF in different subgroups and the significance of the corresponding interaction terms. The dashed reference line indicates odds ratio of 1.0. BMI, body mass index; DM, diabetes mellitus; FPG, fasting plasma glucose; HFimpEF, heart failure with improved ejection fraction; VIM, variability independent of the mean.

Furthermore, subgroup analysis **(** **Figure 2****)** demonstrated interaction terms were non-significant across subgroups of sex, age, BMI, FPG levels, the presence of diabetes and ischemic etiology, indicating the associations between FPG variability and HFimpEF were similar among these subgroups. Especially, the association kept significant irrespective of diabetic conditions and mean FPG levels.

### Sensitivity analyses

Given that diabetic patients, especially those with poor glycemic control, usually have greater glycemic fluctuations, the association between GV and HFimpEF was verified by excluding patients with baseline HbA1c > 8% or on insulin treatment **(** **Supplementary Table 3****)**. We found CV and VIM of FPG persisted to significantly associate with HFimpEF in both models after multivariable adjustment, suggesting that GV was associated with HFimpEF even when patients with poor glycemic control were excluded. Furthermore, a nested case-control study was conducted by matching HFimpEF and persistent HFrEF patients in the cohort at 1:1 ratio **(** **Supplementary Table 4****)**. Consistently, we found patients with high GV (quartile 3~4 had significantly lower probability of HFimpEF than those with low GV (quartile 1~2) assessed by 3 different GV measures **(** **Supplementary Table 5****)**.

## Discussion

The major findings of the present study are that HF patients with higher long-term GV are less likely to experience LV functional improvement. Long-term GV is an independent risk factor for the development of HFimpEF.

Hyperglycemia increases risk of physical impairment (31), coronary heart disease (32), heart failure (33), peripheral artery disease (34) and stroke (35), irrespective of diabetic conditions. Long-term poor glycemic control marked by high HbA1c was associated with higher risk of all-cause mortality and hospitalization for patients with cardiovascular disease (36–39). Recent studies also found that acute hyperglycemic status reflected by stress hyperglycemia ratio predicted adverse outcomes in patients with nonobstructive coronary arteries (40), coronary chronic total occlusion (41) and acute coronary syndrome (42).

Apart from mean glycemic levels, existing evidence reveals that GV, no matter short-term or long-term, is an independent risk factor for the incidence of HF. Of note, both FPG variability and HbA1c variability represent variability of glycemic control levels but comprise different aspects of dysregulated glycemic homeostasis. On one hand, HbA1c, representing a weighted mean glucose level over the preceding 2-3 months, is usually more stable than FPG and thus has less variability (43). On the other hand, HbA1c is an integrated assessment reflecting both FPG and postprandial plasma glucose (PPG) levels (44). A Korean nationwide population-based study revealed that over a median follow-up of 5.3 years, the risk of HF increased by 15% (HR: 1.15 [95% CI 1.10~1.20]) in subjects with the highest quartile of FPG variability compared to those with the lowest quartile (45). A number of diabetic cohort studies demonstrated that higher long-term HbA1c variability valued by different measures was independently associated with increased risk of HF (46–48). In non-diabetic patients, GV accessed by mean amplitude of glycemic excursions (MAGE) was also related to incident HF after myocardial infarction (49). Furthermore, GV has been recognized as a significant predictor for major adverse cardiovascular events (MACE) independent of mean glycemic control levels and conventional risk factors both in diabetic and non-diabetic HF patients (22–25, 50).

Attributed to advanced guideline-directed medical and device therapies, 10%~52% of HF patients have experienced myocardial recovery and developed HFimpEF (1–5). Of note, the specific definition of HFimpEF varies according to different guidelines or clinical studies. The proposed universal definition of HFimpEF (51) put forward a requirement of ≥10-point increase from baseline EF in addition to the criteria of a baseline EF ≤40% and a follow-up measurement > 40% as stated in the 2022 AHA/ACC/HFSA guideline (8). In this study, we adopted the universal definition since a 10-point increase in EF guarantees actual myocardial functional improvement and minimizes the impact by interobserver and intraobserver measurement variabilities.

We recently showed that glucose metabolic disorders reflected by hyperglycemia or insulin resistance are associated with compromised development of HFimpEF (52, 53). However, to our knowledge, the relationship between GV and HFimpEF remains unknown. In accordance with previous reports, 42.0% of hospitalized HF patients in this study developed HFimpEF during 12-month follow-up. Besides, we for the first time demonstrated that LV functional improvement accompanied by reverse remodeling was prominently compromised with increasing long-term FPG variability. Multivariate regression analysis showed that long-term FPG variability was independently associated with the incidence of HFimpEF, even after adjustment for baseline HbA1c as well as mean FPG levels during follow-up. These findings were also confirmed by the nested case-control study. Furthermore, subgroup analysis revealed the association between FPG variability and HFimpEF persisted significant irrespective of the presence of diabetes and mean FPG control levels. In addition, we assessed FPG variability by different measures including CV, ASV and VIM. CV and ASV are relatively simple and more feasible in clinical practice, whereas VIM is calculated based on logarithmic curve fitting to eliminate its correlation with mean FPG. We revealed that all these measures of FPG variability yielded similar findings. These data jointly support the notion that GV *per se* plays a negative role in the development of HFimpEF through mechanisms independent of glycemic levels.

Noteworthy, although only a small proportion of patients were on SGLT2 inhibitors (n=32, 6.2%) since the medication has not been introduced to our center until the second half of 2019, the univariate analysis exhibited a positive association between the use of SGLT2 inhibitors and HFimpEF. SGLT2 inhibitors have pleiotropic cardio-protective effects through modulating renin-angiotensin-aldosterone system, shifting energy substrate, and attenuating systemic inflammatory status (54, 55). Given the promising results from DAPA-HF (56) and EMPEROR-Reduced trials (57), SGLT2 inhibitors have become a cornerstone of HFrEF treatment. Resent trails revealed that SGLT2 inhibitors also improve outcomes of patients with HF with preserved EF, no matter with or without diabetes (58–60). Existing evidence suggested that SGLT2 inhibitors facilitate LV reverse remodeling and diastolic function (61–64). Our results further implied that SGLT2 inhibitors may exert favorable effects on myocardial functional recovery, which certainly awaits further confirmation in prospectively designed clinical studies.

Based on existing clinical and basic studies, several potential mechanisms might account for the negative impact of GV on myocardial recovery. First, dramatic glycemic oscillation promotes oxidative stress in the myocardium, thereby leading to mitochondrial damage, endothelial dysfunction, inflammatory response and finally myocardial fibrosis (65–68). Second, greater GV is presumably associated with more hypoglycemic episodes. In our study, 3.3% of the subjects suffered hypoglycemic event, which was only observed in patients with higher GV. Established evidence has displayed that hypoglycemia stimulates sympathetic nervous system, thus increasing cardiac preload, arrhythmia, inflammation and thereby posing deleterious effects on myocardium (69–71). Third, patients with marked glycemic oscillation tend to have poor compliance to medical treatments, thus attenuating the beneficial effects of pharmacological therapies on myocardial recovery.

### Limitations

Our findings should be interpreted in the context of the following limitations. First, this study is a retrospective analysis based on prospectively collected data from a single center, and the result is potentially subject to selection bias. Second, hypoglycemic episodes were not analyzed and adjusted in the multivariate analysis since they were only documented from long-term FPG values owing to the study design and thus probably underestimated. Third, hospitalization for HF is associated with worsening of EF, which may to some extent affect our findings. Finally, prospective studies are warranted to analyze the causal link between GV and occurrence of HFimpEF.

## Conclusions

In conclusion, our findings suggest that greater long-term GV is associated with compromised development of HFimpEF. A more stable control of glycemic levels might provide favorable effects on myocardial recovery in HF patients even without diabetes.

## Data availability statement

The raw data supporting the conclusions of this article will be made available by the authors, without undue reservation.

## Ethics statement

The studies involving humans were approved by Ruijin Hospital, Shanghai Jiao-Tong University School of Medicine. The studies were conducted in accordance with the local legislation and institutional requirements. The participants provided their written informed consent to participate in this study.

## Author contributions

CY, JC and XW performed study design, data analysis and data interpretation. CY and XW performed manuscript writing. CY, JC, JQ, XS, SF, MA and XW performed data collection. CY, JQ, FD, WS, LL, RZ and XW performed manuscript revision. All authors contributed to the article and approved the submitted version.
