# Supplementary material for: Long-term glycemic variability predicts compromised development of heart failure with improved ejection fraction: a cohort study
Source: Front Endocrinol (Lausanne). 2023 Sep 20;14:1211954. doi: 10.3389/fendo.2023.1211954 (PMC10547879; doi:10.3389/fendo.2023.1211954)
Supplement: Supplementary file 1 [file Table_1.docx]

**Supplementary Table I. Univariate regression analysis for HFimpEF**

| **Variate** | **OR (95% CI)** | ***P*-value** |
| --- | --- | --- |
| Age, per 10 years | 0.760 (0.658~0.875) | <0.001 |
| Sex | 1.420 (0.910~2.243) | 0.127 |
| Non-diabetes | 1.497 (1.018~2.216) | 0.042 |
| Hypertension | 0.895 (0.631~1.269) | 0.532 |
| Atrial fibrillation | 1.063 (0.613~1.826) | 0.824 |
| Smoking habits | 1.303 (0.873~1.948) | 0.196 |
| Body mass index | 1.038 (0.988~1.091) | 0.135 |
| Non-ischemic etiology | 1.509 (1.063~2.145) | 0.021 |
| Systolic BP, per 10 mmHg | 1.117 (1.028~1.216) | 0.010 |
| Diastolic BP, per 10 mmHg | 1.228 (1.091~1.389) | <0.001 |
| HbA1c, per 1% | 0.808 (0.688~0.941) | 0.007 |
| FPG, per mmol/L | 0.993 (0.927~1.062) | 0.846 |
| Triglyceride, per mmol/L | 1.085 (0.915~1.296) | 0.347 |
| Total cholesterol, per mmol/L | 1.146 (0.978~1.345) | 0.093 |
| HDL cholesterol, per mmol/L | 0.886 (0.461~1.689) | 0.714 |
| LDL cholesterol, per mmol/L | 1.199 (0.988~1.459) | 0.067 |
| eGFR, per 10 mL/min/1.732m^2^ | 1.050 (0.964~1.145) | 0.264 |
| NT-proBNP, per SD | 0.930 (0.759~1.124) | 0.461 |
| CRT | 0.770 (0.440~1.317) | 0.347 |
| Beta-blockers | 0.913 (0.544~1.547) | 0.733 |
| ACEI/ARB | 0.942 (0.664~1.335) | 0.737 |
| ARNI | 1.236 (0.837~1.823) | 0.286 |
| SGLT2 inhibitors | 2.115 (1.029~4.478) | 0.044 |
| Spironolactones | 1.293 (0.878~1.917) | 0.196 |
| EF, per 10% | 0.900 (0.647~1.253) | 0.531 |
| EDV index, per 10 mL/m^2^ | 0.916 (0.862~0.971) | 0.004 |
| ESV index, per 10 mL/m^2^ | 0.945 (0.881~1.011) | 0.106 |
| Mean of FPG during follow-up | 0.957 (0.874~1.046) | 0.340 |

ACEI, angiotensin-converting enzyme inhibitors; ARB, angiotensin receptor blockers; ARNI, angiotensin-receptor neprilysin inhibitors; BP, blood pressure; CI, confidence interval; CRT, cardiac resynchronizing therapy; EDV, end-diastolic volume; EF, ejection fraction; eGFR, estimated glomerular filtration rate; ESV, end-systolic volume; FPG, fasting plasma glucose; HDL, high-density lipoprotein; HFimpEF, heart failure with improved ejection fraction; LDL, low-density lipoprotein; NT-proBNP, N-terminal pro-B-type natriuretic peptide; OR, odds ratio; SD, standard deviation; SGLT2, sodium-glucose cotransporter 2.

**Supplementary Table I. Univariate regression analysis for HFimpEF (continue)**

| **Variate** | **OR (95% CI)** | ***P*-value** |
| --- | --- | --- |
| **CV of FPG** |  |  |
| Continuous | 0.160 (0.049~0.490) | 0.002 |
| Categorical |  | 0.002^*^ |
| Q1 (<0.093) | Reference | - |
| Q2 (0.093~0.162) | 1.063 (0.654~1.731) | 0.804 |
| Q3 (0.163~0.268) | 0.630 (0.383~1.033) | 0.068 |
| Q4 (≥0.269) | 0.508 (0.305~0.837) | 0.008 |
| **ASV of FPG** |  |  |
| Continuous | 0.882 (0.794~0.973) | 0.015 |
| Categorical |  | 0.005^*^ |
| Q1 (<0.568) | Reference | - |
| Q2 (0.568~1.190) | 0.669 (0.409~1.089) | 0.107 |
| Q3 (1.191~2.240) | 0.558 (0.339~0.914) | 0.021 |
| Q4 (≥2.241) | 0.499 (0.302~0.819) | 0.006 |
| **VIM of FPG** |  |  |
| Continuous | 0.548 (0.369~0.798) | 0.002 |
| Categorical |  | 0.005^*^ |
| Q1 (<0.408) | Reference | - |
| Q2 (0.408~0.641) | 0.668 (0.408~1.089) | 0.107 |
| Q3 (0.642~0.968) | 0.677 (0.413~1.104) | 0.119 |
| Q4 (≥0.969) | 0.465 (0.280~0.765) | 0.003 |

*P for trend

ASV, average successive variability; CI, confidence interval; CV, coefficient of variation; FPG, fasting plasma glucose; HFimpEF, heart failure with improved ejection fraction; OR, odds ratio; VIM, variability independent of the mean.

**Supplementary Table II. Multivariate regression analysis for HFimpEF (glycemic variability as continuous variable)**

|  | **Model 1** | | **Model 2** | | **Model 3** | | **Model 4** | |
| --- | --- | --- | --- | --- | --- | --- | --- | --- |
|  | **OR (95% CI)** | ***P*** | **OR (95% CI)** | ***P*** | **OR (95% CI)** | ***P*** | **OR (95% CI)** | ***P*** |
| **CV of FPG** | 0.226 (0.063~0.762) | 0.019 | 0.126 (0.027~0.535) | 0.006 | 0.132 (0.028~0.575) | 0.008 | 0.226 (0.063~0.762) | 0.019 |
| **ASV of FPG** | 0.896 (0.806~0.990) | 0.035 | 0.908 (0.808~1.012) | 0.090 | 0.830 (0.705~0.969) | 0.021 | 0.832 (0.705~0.974) | 0.025 |
| **VIM of FPG** | 0.572 (0.382~0.839) | 0.005 | 0.584 (0.387~0.865) | 0.009 | 0.575 (0.376~0.861) | 0.009 | 0.579 (0.377~0.872) | 0.011 |

Model 1, adjustment for age and sex;

Model 2, additional adjustment for HF etiology, systolic and diastolic blood pressure, body mass index and history of diabetes;

Model 3, additional adjustment for HbA1c, renal function, mean fasting glucose levels during follow-up, and baseline left ventricular end-diastolic volume index;

Model 4, additional adjustment for CRT, use of beta-blockers, angiotensin converting enzyme inhibitors, angiotensin receptor blockers, angiotensin receptor-neprilysin inhibitors, spironolactones, sodium-glucose cotransporter 2 inhibitors.

ASV, average successive variability; CI, confidence interval; CRT, cardiac resynchronizing therapy; CV, coefficient of variation; FPG, fasting plasma glucose; HF, heart failure; HFimpEF, heart failure with improved ejection fraction; OR, odds ratio; VIM, variability independent of the mean.

**Table III. Sensitivity analysis**

| **GV**  **measures** | **Model I** | | **Model II** | | |  |
| --- | --- | --- | --- | --- | --- | --- |
|  | **OR (95% CI)** | ***P*** | | **OR (95% CI)** | ***P*** | |
| **CV** | 0.132 (0.024~0.670) | 0.017 | | 0.079 (0.013~0.422) | 0.004 | |
| **ASV** | 0.850 (0.702~1.022) | 0.090 | | 0.778 (0.637~0.940) | 0.011 | |
| **VIM** | 0.590 (0.376~0.903) | 0.018 | | 0.550 (0.343~0.862) | 0.011 | |

All the above models were adjusted for age, sex, HF etiology, systolic and diastolic blood pressure, body mass index and history of diabetes, CRT, use of beta-blockers, angiotensin converting enzyme inhibitors, angiotensin receptor blockers, angiotensin receptor-neprilysin inhibitors, spironolactones, sodium-glucose cotransporter 2 inhibitors.

Model I, excluding patients with HbA1c > 8%;

Model II, excluding patients on insulin treatment.

ASV, average successive variability; CI, confidence interval; CRT, cardiac resynchronizing therapy; CV, coefficient of variation; GV, glycemic variability; HF, heart failure; OR, odds ratio; VIM, variability independent of the mean.

^*^*P* for trend.

**Supplementary Table IV. Baseline demographic and clinical characteristics of persistent HFrEF and HFimpEF patients in the nested case-control study**

|  | **HFrEF** | **HFimpEF** | ***P*-value** |
| --- | --- | --- | --- |
| n | 200 | 200 |  |
| ***Demographic characteristics and clinical assessments*** | | | |
| Male sex | 172 (86.0) | 172 (86.0) | 1.000 |
| Age, years | 59.53±13.73 | 59.45±13.87 | 0.957 |
| Diabetes | 68 (34.0) | 53 (26.5) | 0.128 |
| Hypertension | 121 (60.5) | 105 (52.5) | 0.130 |
| Atrial fibrillation | 21 (10.5) | 24 (12.0) | 0.752 |
| Dyslipidemia | 139 (69.5) | 128 (64.0) | 0.289 |
| Smoking habits | 94 (47.0) | 88 (44.0) | 0.616 |
| BMI, kg/m^2^ | 25.59±4.42 | 24.88±3.67 | 0.089 |
| Systolic BP, mmHg | 128.18±24.04 | 127.35±24.96 | 0.741 |
| Diastolic BP, mmHg | 77.41±16.49 | 78.26±17.27 | 0.621 |
| Ischemic etiology | 112 (56.0) | 99 (49.5) | 0.229 |
| NYHA grades  (II/III/IV) | 40 (20.0) / 133 (66.5) / 27 (13.5) | 43 (21.5) / 124 (62.0) / 33 (16.5) | 0.599 |
| ***Laboratory measurements*** | | | |
| HbA1c, % | 6.60±1.34 | 6.29±1.08 | 0.012 |
| FPG, mmol/L | 5.58 (4.79~6.83) | 5.46 (4.92~6.82) | 0.743 |
| Triglyceride, mmol/L | 1.21 (0.96~1.64) | 1.35 (1.02~1.75) | 0.097 |
| Total cholesterol, mmol/L | 3.93±1.21 | 4.12±1.07 | 0.103 |
| HDL cholesterol, mmol/L | 0.99±0.24 | 1.04±0.26 | 0.063 |
| LDL cholesterol, mmol/L | 2.42±0.91 | 2.54±0.90 | 0.183 |
| eGFR, mL/min/1.732m^2^ | 89.17±23.37 | 88.54±16.65 | 0.756 |
| NT-proBNP, pg/mL | 2214.0 (996.1~4954.0) | 2411.5 (933.5~4947.3) | 0.939 |
| ***CRT implantation*** | | | |
| CRT | 30 (15.0) | 20 (10.0) | 0.174 |
| ***Medication*** | | | |
| Aspirin | 108 (54.0) | 109 (54.5) | 1.000 |
| P_2_Y_12_ inhibitors | 94 (47.0) | 87 (43.5) | 0.547 |
| Beta-blockers | 184 (92.0) | 175 (87.5) | 0.187 |
| ACEI/ARB | 122 (61.0) | 97 (48.5) | 0.016 |
| ARNI | 47 (23.5) | 62 (31.0) | 0.116 |
| SGLT2 inhibitors | 6 (3.0) | 19 (9.5) | 0.013 |
| Spironolactones | 151 (75.5) | 149 (74.5) | 0.908 |
| Diuretics | 143 (71.5) | 130 (65.0) | 0.197 |
| Statins | 117 (58.5) | 102 (51.0) | 0.160 |

ACEI, angiotensin-converting enzyme inhibitors; ARB, angiotensin receptor blockers; ARNI, angiotensin-receptor neprilysin inhibitors; BMI, body mass index; BP, blood pressure; CRT, cardiac resynchronizing therapy; eGFR, estimated glomerular filtration rate; FPG, fasting plasma glucose; HDL, high-density lipoprotein; HFimpEF, heart failure with improved ejection fraction; HFrEF, heart failure with reduced ejection fraction; LDL, low-density lipoprotein; NT-proBNP, N-terminal pro-B-type natriuretic peptide; NYHA, New York Heart Association; SGLT2, sodium-glucose cotransporter 2.

**Supplementary Table V. Multivariate regression analysis for development of HFimpEF by dichotomized glycemic variability in the nested case-control study**

| **GV**  **measures** | **Model 1** | | **Model 2** | | | **Model 3** | | |  |
| --- | --- | --- | --- | --- | --- | --- | --- | --- | --- |
|  | **OR (95% CI)** | ***P*** | | **OR (95% CI)** | ***P*** | | **OR (95% CI)** | ***P*** | |
| **CV** | 0.440 (0.267~0.725) | 0.001 | | 0.332 (0.184~0.600) | <0.001 | | 0.307 (0.166~0.568) | <0.001 | |
| **ASV** | 0.546 (0.330~0.903) | 0.018 | | 0.446 (0.246~0.810) | 0.008 | | 0.438 (0.237~0.812) | 0.009 | |
| **VIM** | 0.563 (0.361~0.879) | 0.012 | | 0.518 (0.314~0.855) | 0.010 | | 0.483 (0.287~0.813) | 0.006 | |

Model 1, adjustment for HF etiology, systolic and diastolic blood pressure, body mass index and history of diabetes;

Model 2, additional adjustment for HbA1c, renal function, mean fasting glucose levels during follow-up, and baseline left ventricular end-diastolic volume index;

Model 3, additional adjustment for CRT, use of beta-blockers, angiotensin converting enzyme inhibitors, angiotensin receptor blockers, angiotensin receptor-neprilysin inhibitors, spironolactones, sodium-glucose cotransporter 2 inhibitors.

ASV, average successive variability; CI, confidence interval; CRT, cardiac resynchronizing therapy; CV, coefficient of variation; GV, glycemic variability; HF, heart failure; HFimpEF, heart failure with improved ejection fraction; OR, odds ratio; VIM, variability independent of the mean.
